# Pyroptosis in epilepsy: from pathophysiological mechanisms to therapeutic strategies

**DOI:** 10.3389/fcell.2026.1793636

**Published:** 2026-04-24

**Authors:** Qun Chen, Yu-Tao Yang, Li Cai, Hai-Qing Zhang, Juan Yang

**Affiliations:** 1 Department of Neurology, The Affiliated Hospital of Zunyi Medical University, Zunyi, Guizhou, China; 2 Key Laboratory of Brain Function and Brain Disease Prevention and Treatment of Guizhou Province, Zunyi, China

**Keywords:** epilepsy, pyroptosis, blood–brain barrier, neurons, glial cells, targeted therapy

## Abstract

Epilepsy affects millions of individuals worldwide, however, approximately one-third of patients exhibit pharmacoresistance to currently available antiseizure therapies, underscoring an urgent unmet need for novel mechanism-based therapeutic strategies. Pyroptosis, a Gasdermin-mediated proinflammatory cell death, has recently been implicated as a pivotal driver of epileptogenesis and disease progression. This narrative critically evaluates emerging preclinical and clinical evidence linking pyroptotic signaling to epilepsy pathophysiology, with particular attention to cell-type-specific contributions. In astrocytes, GSDMD activation compromises blood–brain barrier (BBB) integrity, an effect mediated by the downregulation of endothelial tight junction proteins. In neurons, activation of the TRPM7/ROS/JAK2/STAT3 pathway drives pyroptosis, a process that also involves the interaction between NLRP3 and mitophagy. Microglial pyroptosis amplifies neuroinflammation, creating a self-perpetuating cycle. Clinically, the caspase-1 inhibitor VX-765 demonstrated favorable safety and preliminary efficacy in a Phase II randomized controlled trial in patients with refractory epilepsy; however, the trial did not meet its primary efficacy endpoint, suggesting that longer treatment durations may be required to assess its therapeutic potential. Preclinically, the GSDMD inhibitor disulfiram shows BBB-protective effects but is limited by off-target hepatotoxicity; conversely, development of the NLRP3 inhibitor MCC950 was discontinued following adverse hepatic findings in early clinical studies. Accumulating evidence also suggests that non-canonical pyroptotic pathways (caspase-4/5/11, GSDME) and PANoptosis, which integrates pyroptosis, apoptosis, and necroptosis, play a role in epileptic neuronal death. Major translational hurdles poor CNS bioavailability of candidate inhibitors, compensatory activation of parallel cell death pathways, and the absence of validated, clinically actionable biomarkers. Future efforts should therefore focus on developing cell-selective pyroptosis modulators, advanced CNS-targeted delivery platforms (e.g., nanoparticle- or antibody-conjugated systems), and biomarker-informed patient enrichment strategies to enable rational clinical translation of pyroptosis-directed therapeutics.

## 1 Introduction

Epilepsy is a common chronic neurological disorder characterized by transient, recurrent and stereotyped seizures. The pathological mechanism involves multiple factors. The traditional view primarily focuses on abnormal neuronal discharges resulting from aberrant ion channels function and an imbalance between excitation and inhibition (E/I). However, an growing body of evidence indicates that the occurrence and development of epilepsy is a complex network process involving neuroinflammation, immune activation, blood–brain barrier (BBB) disruption, and various programmed cell death patterns. Recent studies have demonstrated that neuroinflammation can lead to neuronal network anomalies by affecting glial cells and immune cells within the brain. For example, activated microglia and astrocytes release inflammatory factors that disrupt the balance between excitation and inhibition in neural circuits. Which in turn suppress endogenous antiepileptic systems, thereby contributing to the development of drug-resistant epilepsy (Bhat et al., 2021; Vega García et al., 2025; Shi et al., 2025). Despite significant progress made in basic and clinical research, the underlying pathological mechanisms remains unclear in approximately 60% of epilepsy, and nearly one-third of them suffer from drug-resistant epilepsy, which severely impairs their quality of life (Li et al., 2023). Therefore, in-depth investigation of novel molecular mechanisms in epilepsy and the development of corresponding targeted therapeutic interventions have become critical research priorities in the field (Asadi-Pooya et al., 2023).

**FIGURE 1:**
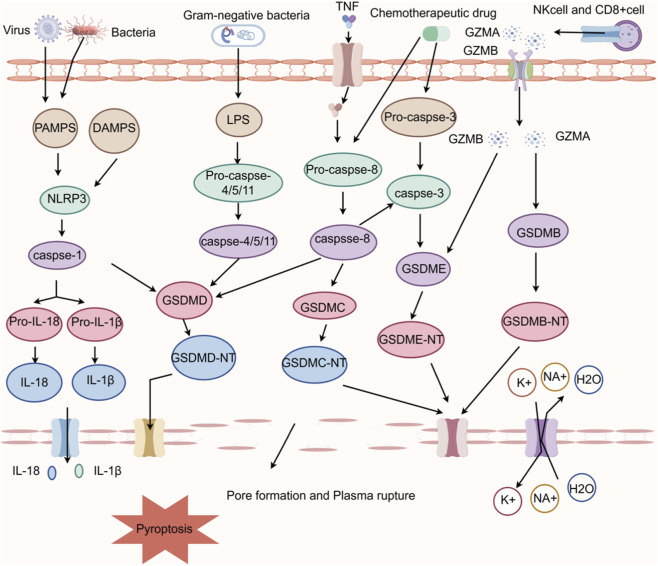
Key signaling pathways involved in pyroptosis. Pyroptosis is primarily mediated by gasdermin family proteins through two major pathways. In the canonical pathway, damage-associated molecular patterns (DAMPs) or pathogens-associated molecular patterns (PAMPs) trigger inflammasome assembly- such as NLRP3inflammasome- which recruits and activates caspase-1. Activated caspase-1 cleaves GSDMD, release its N-terminal pore-forming domain. These fragments oligomerize and insert into the plasma membrane to form pores, ultimately leading to osmotic lysis. Concurrently, caspase-1 processes pro-IL-1β and pro-IL-18 into their bioactive, mature forms. In the non-canonical pathway, intracellular lipopolysaccharide (LPS) directly activates human caspase-4 or caspase-5 -or murine caspase-11, which then cleave GSDMD to induce pore formation Additional gasdermin members contributes to pyroptotic regulation: caspase-8 and caspase-3-mediated cleavage of GSDMD or GSDME can lead to pyroptosis; granzyme A (GzmA) cleaves GSDMB, and granzyme B (GzmB) also cleaves GSDME. Collectively, these pathways constitute a highly interconnected regulatory network governing pyroptosis (Created with Figdraw).

Pyroptosis, a form of programmed inflammatory cell death mediated by the Gasdermin protein family, has garnered increasing attention in recent years for its role in central nervous system (CNS) disorders (Chen et al., 2023; Oladapo et al., 2024). Unlike apoptosis, pyroptosis relies on inflammasomes (such as NLRP3) to activate caspase-1 or caspase-4/5/11, which cleave and activate the key effector protein gasdermin D (GSDMD). Its N-terminal domain (GSDMD-NT)translocates to the cell membrane to form pores, facilitating the release of inflammatory mediators such as IL-1β and IL-18, which cause plasma membrane rupture and initiate a potent inflammatory cascade (Rao et al., 2022; Wei et al., 2022; Pei et al., 2023; Yu et al., 2021). Beyond this classical pathway, non-canonical mechanisms—such as caspase-3/8 -mediated cleavage of GSDME/GSDMC and granzyme A (GzmA) -mediated cleavage of GSDMB—have been progressively identified, collectively forming a complex pyroptosis regulatory network (as illustrated in Figure 1) (Yang et al., 2015; Hara et al., 2024; Ma and Lieberman, 2024; Heinke, 2025; Zhong et al., 2025; Zhong et al., 2023; Zhou et al., 2020). In recent years, studies of surgical specimens from patients with temporal lobe epilepsy, as well as various animal models of epilepsy—including those induced by KA and pilocarpine—have revealed activation of the NLRP3 inflammasome and increased expression of the GSDMD-NT fragment. Furthermore, epilepsy severity has been found to correlate positively with IL-1β levels (Wu et al., 2025; Sun W. et al., 2023; Yu et al., 2024; Xu et al., 2025), suggesting pyroptosis may serve as a pivotal bridge linking seizures, neuroinflammation, and neuronal damage in epilepsy pathogenesis. Seizures can cause neuronal injury in vulnerable brain regions such as the hippocampus, potentially triggering pyroptosis. Subsequently, the release of inflammatory factors such as IL-1β further activates microglia and astrocytes, thereby amplifying neuroinflammation. Such inflammatory microenvironments lower neuronal excitability thresholds, impede neural repair, and promote aberrant synaptic remodeling. This establishes a deleterious feedback loop: seizures induce pyroptosis and inflammation, which in turn increase seizure susceptibility.Over time, this loop contributes to the formation of epileptic foci and disease progression (Yang et al., 2022; Ge et al., 2024; Du et al., 2022; McKenzie et al., 2020; Atabaki et al., 2023). Although some studies suggest that moderate or tightly regulated pyroptosis may help maintain neural network stability by eliminating severely damaged neurons-cells that might otherwise evolve into aberrant seizure foci (Hao and Feng, 2023; Hu et al., 2022; Xue et al., 2022)- the majority of evidence indicates that excessive pyroptosis tends to promote epileptogenesis.

Given the emerging significance of pyroptosis in epilepsy, this narrative review synthesizes current knowledge on its pathophysiological mechanisms, therapeutic targeting strategies, and future research directions. It aims to provide innovative and clinically translatable theoretical foundations for the development of novel anti-epileptic interventions.

## 2 The role of pyroptosis in the pathophysiology of epilepsy

**FIGURE 2:**
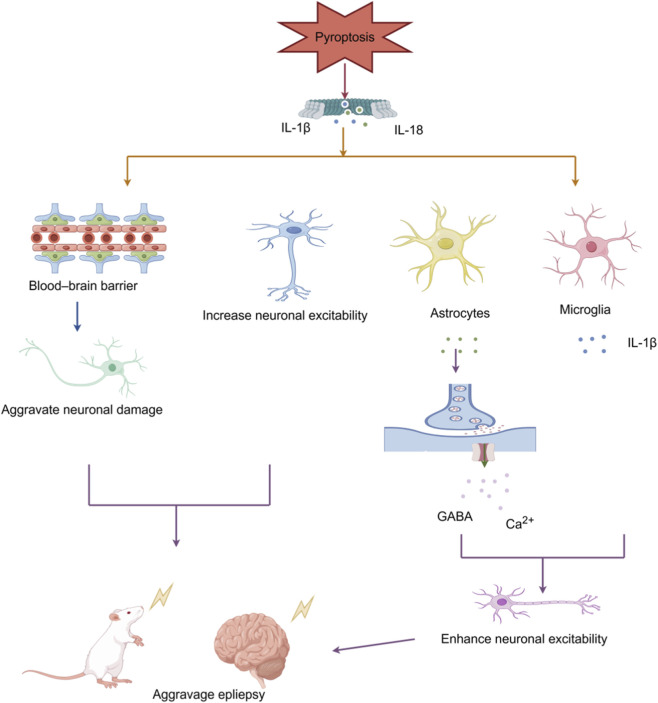
The role of pyroptosis in the pathogenesis of epilepsy. Pyroptosis releases inflammatory cytokines such as IL-1β and IL-18, which directly enhance neuronal excitability while simultaneously disrupting the blood–brain barrier and exacerbating neuronal damage. Furthermore, it activates microglia, triggering the release of additional inflammatory cytokines. It also increases neuronal excitability by modulating neurotransmitter transmission and promoting calcium influx via astrocyte disfunction, thereby aggravating epileptic seizures (Created with Figdraw).

Pyroptosis influences the function of the Blood–Brain Barrier, neurons, and glial cells through multiple mechanisms, playing a significant role in the pathophysiological processes underlying epilepsy. It not only directly induces neuronal death but also activates immune cells via the release of inflammatory cytokines, thereby establishing a vicious cycle that perpetuates neuroinflammation and drives epileptogenic pathological damage (See Figure 2 for details).

### 2.1 Impairment of the blood–brain barrier

The blood–brain barrier plays a vital role in maintaining the stability and health of the CNS. Studies have found that dysfunction of the blood–brain barrier is closely linked to epileptic seizures. At the same time, research in animal models has shown that seizures themselves can damage this critical barrier (Bar-Klein et al., 2017; Reiter et al., 2024). Zhang M. et al. (2025) demonstrated that astrocyte pyroptosis directly contributes to blood–brain barrier disruption in a KA-induced murine model of epilepsy. Their findings showed that following seizures, mice exhibited significant blood–brain barrier leakage and increased expression of pyroptosis-associated markers—including NLRP3, caspase-1, and GSDMD-NT—in hippocampal tissue. Immunofluorescence co-localization analysis revealed that GSDMD cleavage occurred predominantly in GFAP-positive astrocytes, not in NeuN-positive neurons, suggesting that astrocytes are the primary cell type undergoing pyroptosis in epilepsy. *In vitro* experiments further explored the glia–endothelium interaction. When conditioned medium from astrocytes treated with LPS and ATP (to induce pyroptosis) was applied to cultures of cerebral microvascular endothelial cell, a marked reduction was observed in the expression of tight junction proteins—including ZO-1, claudin-5, and occludin. However, when astrocytes were pretreated with disulfiram, a GSDMD inhibitor, this detrimental effect on endothelial tight junctions was significantly attenuated. Together, these results directly demonstrate that astrocytic pyroptosis compromises blood–brain barrier integrity by releasing inflammatory mediators that suppress the expression of tight junction protein in endothelial cells (Zhang M. et al., 2025; Xia L. et al., 2021). Recent work by Zhou et al. (2026) has further elucidated the molecular mechanism linking astrocytic pyroptosis to neuronal dysfunction in epilepsy. Using a KA-induced murine epilepsy model, they demonstrated that seizure activity upregulates astrocytic Lipocalin-2 (LCN2), which triggers the NLRP3 inflammasome and leads to GSDMD-mediated pyroptosis. Interestingly, the same LCN2/NLRP3 axis also inhibits the transfer of mitochondria from astrocytes to stressed neurons via tunneling nanotubes (TNTs)—a neuroprotective process under physiological conditions Inhibiting this pathway, either genetically or pharmacologically, not only prevented astrocytic pyroptosis but also enhanced mitochondrial transfer, leading to improved neuronal bioenergetics and neurological outcomes. Consequently, neurons received enhanced metabolic support, and overall neurological function improved. These findings reveal that LCN2 acts as a key molecular hub, linking inflammatory cell death with intercellular mitochondrial rescue processes, thereby establishing a mechanistic link between glial pyroptosis and neuronal dysfunction in epilepsy (Zhou et al., 2026).

### 2.2 Effects on neuronal excitability

Pyroptosis not only directly leads to neuronal death, but also alters the excitability and function of surviving neurons through various mechanisms, these effects constitute a core pathological substrate underlying epileptogenesis.

#### 2.2.1 Direct cytotoxicity of GSDMD

Substantial experimental evidence supports GSDMD-mediated neuronal pyroptosis in epilepsy models. Xia et al. (2022) reported, through integrated bioinformatic analysis and experimental validation, that in a murine model of status epilepticus induced by KA, hippocampal tissue exhibits significant upregulation of both GSDMD mRNA and protein expression. Concurrently, alongside robust accumulation of the GSDMD-NT following seizure onset—indicating caspase 1-dependent proteolytic cleavage and activation of GSDMD (Xia et al., 2022). At the same time, they found that quantitative RT–PCR revealed no significant alteration in GSDME mRNA abundance between KA-treated and control animals. However, the WB results showed that the expression of full-length GSDME was decreased and GSDME-N-terminus were significantly increased after SE. Collectively, these data provide direct mechanistic evidence for GSDME-driven pyroptotic neuronal death in epilepsy. Notably, while these findings confirm seizure-associated GSDME processing, they do not establish causal involvement of GSDME in neuronal injury. Targeted loss-of-function (e.g., conditional GSDMD knockout) or gain-of-function (e.g., astrocyte-specific GSDMD overexpression) approaches are required to establish this link. Complementing these observations, a review by Atabaki et al. (2023) synthesized convergent evidence from rodent epilepsy models—including KA- and pilocarpine-induced temporal lobe epilepsy—as well as human resected hippocampal tissue from patients with drug-resistant temporal lobe epilepsy and hippocampal sclerosis, reporting consistent detection of caspase-1 activation and GSDMD cleavage. Mechanistically, GSDMD-NT oligomerizes within the neuronal plasma membrane to form pores, thereby disrupting ion homeostasis—specifically promoting K^+^efflux and Ca^2+^influx—and inducing osmotic imbalance. This cascade culminates in oncotic swelling, plasma membrane rupture, and lytic neuronal death (Atabaki et al., 2023).

#### 2.2.2 Mitochondrial dysfunction indirectly triggers neuronal hyperexcitability

Emerging evidence indicates that pyroptosis is closely associated with mitochondrial function (Mishra et al., 2021; Miao et al., 2023). Pyroptosis can directly impair mitochondria integrity, resulting in mitochondrial dysfunction, dysregulated cellular energy metabolism, and elevated reactive oxygen species (ROS) generation. Concurrently, activation of the upstream NLRP3 inflammasome during pyroptosis suppresses mitophagy, resulting in the accumulation of dysfunctional mitochondria and further amplifying ROS production. This ROS surge, in turn, reinforces NLRP3 inflammasome activation, establishing a self-amplifying pathological loop that perpetuates pyroptosis and enhances neuronal excitability (Wu et al., 2025; Billingham et al., 2022). Notably, STAT3 has been shown to transcriptionally upregulate NLRP3 expression by directly binding to its promoter region; this interaction promotes histone H3 lysine 9 acetylation (H3K9ac), thereby enhancing chromatin accessibility and driving NLRP3/caspase-1-mediated neuronal pyroptosis and exacerbating neuronal damage in epileptic mice (Jiang et al., 2021). Furthermore, Tong et al. (2024) demonstrated that in a pilocarpine-induced mice model of epilepsy, neuronal TRPM7 channel expression is significantly upregulated. As TRPM7 function as a Zn^2+^-permeable cation channel, its overexpression leads to substantial intracellular Zn^2+^ influx and, pathological Zn^2+^ accumulation. This Zn^2+^ overload triggers mitochondrial oxidative stress and markedly increases mitochondrial ROS production. Elevated ROS subsequently activates the JAK2/STAT3 signaling pathway which in turn potentiates the expression and activation of the NLRP3 inflammasome—culminating in pyroptotic neuronal death. This process contributes to the development and progression of epilepsy (Tong et al., 2024). Critically, this cascade not only executes caspase-dependent cell death but also induces a pre-lytic reduction in the neuronal excitation threshold. As a result, neurons exhibit heightened responsiveness to synaptic inputs and increased propensity for aberrant, synchronized firing—providing a key electrophysiological substrate for epileptiform discharges (Vasudevan et al., 2023).

In summary, pyroptosis contributes to neuronal loss and network hyperexcitability in epilepsy through multiple interrelated mechanisms—including plasma membrane rupture, disruption of ionic homeostasis, and mitochondrial failure. Collectively, these effects drive progressive neuronal depletion within epileptic foci and foster the emergence of pathologically synchronized neuronal ensembles. Thus, pyroptosis serves as a pivotal and non-redundant pathogenic driver in epileptogenesis and disease progression.

#### 2.2.3 PANoptosis: an integrated inflammatory cell death pathways in epilepsy

Recent studies suggest that neuronal death in epilepsy arises not from a single programmed cell death modality, but rather from coordinated crosstalk among multiple inflammatory and lytic pathways. PANoptosis—recently conceptualized as a holistic, innate immune–driven cell death paradigm—represents the simultaneous engagement and molecular integration of pyroptotic, apoptotic, and necroptotic effectors within individual neurons or local neural circuits. This integrated process is orchestrated by multiprotein PANoptosome complexes that concurrently activate caspase-1 (driving gasdermin D–mediated pore formation), caspase-8 (initiating apoptotic cleavage cascades), and RIPK3–MLKL (inducing plasma membrane rupture), thereby synergistically amplifying inflammation, loss of cellular integrity, and irreversible neuronal demise (Samir et al., 2020).

##### 2.2.3.1 Bioinformatics evidence

Liu et al. (2024) systematically investigated the role of PANoptosis in epileptic pathogenesis for the first time by integrating bioinformatic analyses with experimental validation. Leveraging hippocampal gene expression profiles from KA–induced epileptic mice deposited in the Gene Expression Omnibus (GEO) database (GSE99577), the study employed a curated PANoptosis-related gene signature—compiled from GeneCards, peer-reviewed literature, and established molecular criteria—to perform Gene Set Variation Analysis (GSVA), Weighted Gene Co-expression Network Analysis (WGCNA), and differential expression analysis. GSVA revealed significant enrichment of PANoptosis-associated transcriptional programs in KA-treated mice relative to controls, demonstrating robust discriminative capacity between epileptic and non-epileptic states. WGCNA identified a highly epilepsy-correlated blue module, within which ten hub genes—MLKL, IRF1, RIPK1, GSDMD, CASP1, CASP8, ZBP1, CASP6, PYCARD, and IL18—were prioritized as candidate biomarkers.

The researchers confirmed the expression patterns of 10 marker genes in human blood samples, animal models, and cell models using Western blot and Quantitative RT–PCR. The study suggests that hippocampal neuronal death in epilepsy may be closely associated with PANoptosis; however, the authors explicitly state that this constitutes only correlational evidence, and the causal role of PANoptosis requires further experimental validation (Liu et al., 2024).

##### 2.2.3.2 Functional evidence

Sun et al. (2025) provided direct experimental evidence supporting PANoptosis-like neuronal death in a murine model of pilocarpine-induced status epilepticus. Their study first identified a significant upregulation of Pannexin-1 (Panx1) expression in the serum of patients with epilepsy and in the hippocampus of pilocarpine-induced epileptic mice. Subsequent administration of the Panx1 inhibitor probenecid ameliorated epileptiform EEG activity, attenuated cognitive deficits, reduced hippocampal neuronal loss, and suppressed molecular signatures of PANoptosis. Pharmacological inhibition experiments further revealed that selective inhibition of pyroptosis, apoptosis, or necroptosis alone conferred only partial protection against muscarinic receptor–mediated neuronal death, whereas concurrent inhibition of all three pathways yielded significantly enhanced and synergistic neuroprotection—demonstrating that neuronal death in this model results from the integrated activation of multiple cell death pathways. Consistent with this, Western blot analysis demonstrated upregulation of PANoptosome-associated proteins—including ZBP1, ASC, caspase-1, caspase-8, RIPK1, RIPK3—as well as dysregulation of canonical effectors of pyroptosis (e.g., cleaved GSDMD), apoptosis (e.g., cleaved caspase-3), and necroptosis (e.g., phosphorylated MLKL), accompanied by ultrastructural hallmarks of inflammatory cell death. Collectively, these functional data establish Panx1 as a critical upstream regulator and validate PANoptosis as a convergent execution mechanism in acute acquired epilepsy. Nevertheless, current evidence remains largely confined to acute chemoconvulsant models (pilocarpine and KA); the spatiotemporal dynamics, cell-type specificity (e.g., neurons versus astrocytes versus microglia), and pathogenic relevance of PANoptosis in chronic, genetic, or post-traumatic forms of epilepsy require systematic investigation (Sun et al., 2025).

### 2.3 Disruption of glial cell homeostasis and function

The release of proinflammatory mediators—including IL-1β, IL-18, and DAMPs (such as HMGB1)—during pyroptosis does not occur in isolation. Instead, these molecules function collectively as intercellular signaling effectors that actively engage neighboring glial cells (astrocytes and microglia).Consequently, pyroptosis-driven glial dysregulation amplifies neuroinflammation, disrupts neuronal network stability, and contributes to progressive neuropathology.

#### 2.3.1 Astrocyte dysfunction and homeostatic collapse

Astrocytes—the most abundant glial cell type in the CNS—serve as indispensable guardians of neuronal homeostasis. They provide metabolic support to neurons via lactate and glutamine shuttling, scavenge neurotransmitters (e.g., glutamate) to ensure normal synaptic transmission, regulate extracellular ionic balance (notably K^+^ buffering) and pH, and release neurotrophic factors (Augusto-Oliveira et al., 2020; Lee et al., 2022). Besides, they regulate synaptic structure and function, promoting new synapse formation and removing dysfunctional synapses via phagocytosis to maintain neural network stability (Vainchtein and Molofsky, 2020). However, within the proinflammatory milieu driven by pyroptosis, astrocytes undergo functional dysregulation and reactive proliferation, culminating in systemic disruption of the glutamate–glutamine cycle and broader homeostatic failure. Specific mechanisms include: (i) Proinflammatory cytokines—including IL-1β and TNF-α—directly suppress the expression and function of excitatory amino acid transporter 2 (EAAT2/GLT-1) in astrocytes, leading to impaired glutamate clearance from the synaptic cleft, pathological accumulation of extracellular glutamate and consequent overactivation of neuronal NMDA and AMPA receptors. This cascade initiates excitotoxic neuronal injury—a core mechanism underpinning neuronal hyperexcitability and death in epilepsy (Xia L. et al., 2021; Rimmele et al., 2021; Long et al., 2023; Hermanova et al., 2024). (ii) Reactive astrocytic proliferation disrupts K^+^ and water homeostasis via coordinated downregulation of the inwardly rectifying potassium channel Kir4.1 and mislocalization/dysfunction of the astrocytic aquaporin channel AQP4. This dual deficit promotes extracellular K^+^accumulation and lowering the seizure threshold (Hermanova et al., 2024). (iii) Sustained inflammation further dysregulates astrocytic glutamate metabolism: reduced glutamate uptake diminishes substrate availability for the tricarboxylic acid (TCA) cycle, leading to bioenergetic failure, mitochondrial depolarization, and elevated reactive oxygen species (ROS) production—thereby exacerbating oxidative stress and neuronal vulnerability (Vezzani et al., 2022). (iv) Critically, activated astrocytes actively release inflammatory mediators, contributing to the maintenance of an inflammatory microenvironment and perpetuating a vicious cycle (Escartin et al., 2021). Collectively, pyroptosis-triggered astrocyte dysfunction represents a pivotal amplifier—not merely a passive responder—in the epileptogenic cascade.

#### 2.3.2 Microglial activation and neuroinflammatory amplication

As the primary resident immune cells of the CNS, microglia serve as critical sentinels and immunoregulators—constituting the first line of immune defence in the CNS against pathological insults. Pyroptosis exacerbates neuroinflammation through multifaceted microglial activation mechanisms. First, pyroptotic neurons and reactive astrocytes release IL-1β—a key cytokine that promotes microglial polarisation toward the proinflammatory M1 phenotype (Zubillaga et al., 2023; Yang et al., 2023). Upon M1 polarization, microglia upregulate and release multiple inflammatory mediators, including IL-1β, TNF-α, IL-12 and inducible nitric oxide synthase (iNOS, inducing excessive nitric oxide production), and activate the NF-κB pathway, which in turn induces caspase-3 expression and contributes to neuronal degeneration. Second, DAMPs, such as ATP and HMGB1, released from pyroptotic cells engage pattern recognition receptors on microglia: ATP binds P2X7 receptors, while HMGB1 signals via TLR4. This dual engagement triggers NLRP3 inflammasome assembly, and also triggers pyroptosis in the microglia (Liu et al., 2022; Pelegrin, 2021). Consequently, activated microglia undergo lytic cell death, releasing additional IL-1β, IL-18 and DAMPs, thereby forming a self-perpetuating cycle of inflammatory amplification. Third, chronic M1 polarization impairs the phagocytic capacity of microglia, prevents effective clearance of damaged neurons and abnormal synaptic connections. And exacerbates neural network dysfunction and maintains the inflammatory microenvironment. Finally, large-scale release of inflammatory cytokines and chemokines during pyroptosis establishes a robust positive feedback loop that significantly accelerates epileptic disease progression: microglial activation begets further inflammatory mediators secretion, which in turn amplifies microglial reactivity and sustains the neuroinflammatory milieu (Long et al., 2023; Purnell et al., 2023). Notably, selectively knocking out GSDMD in microglia disrupts their functional equilibrium—shifting the balance toward heightened proinflammatory signaling while concurrently suppressing phagocytic capacity—thereby exacerbating neuroinflammation and seizure severity (Lin et al., 2023).

In summary, pyroptosis significantly intensifies neuroinflammatory responses by impairing astrocyte function and activating microglia, thereby establishing a mechanistic bridge between innate immune dysregulation and epileptic pathophysiology.

## 3 Potential therapeutic strategies targeting pyroptosis in epilepsy

**FIGURE 3:**
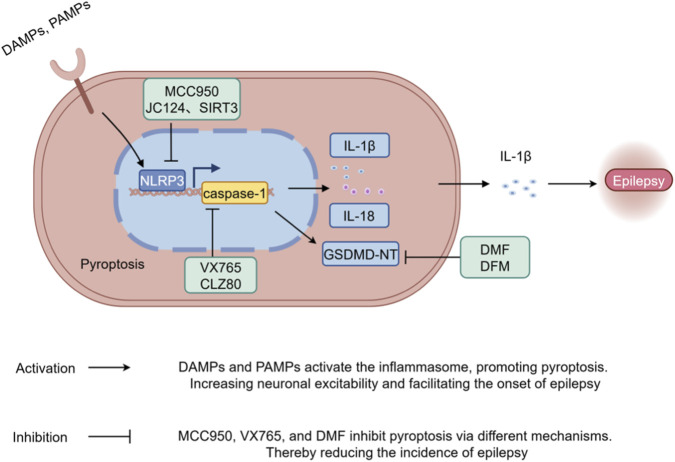
Activation mechanisms and therapeutic intervention points of the pyroptotic signaling cascade in epilepsy. Pattern recognition receptors (such as NLRP3) activate the pro-caspase-1; subsequently, caspase-1 further cleaves pro-IL-1β, pro-IL-18,and full-length GSDMD, generating mature IL-1β, IL-18, and GSDMD-NT. GSDMD-NT oligomerizes at the plasma membrane to form selective ion-permeable pores, facilitating the release of mature inflammatory cytokines, including IL-1β and IL-18. This lytic process amplifies neuroinflammation, promotes neuronal hyperexcitability and contributes to seizure exacerbation and epileptogenesis. Pharmacological inhibitors—including the NLRP3-specific antagonist MCC950, the caspase-1 inhibitor VX-765 and GSDMD inhibitor Disulfiram (DFM)—target distinct nodes of this pathway: MCC950 suppresses inflammasome priming and assembly, whereas VX-765 directly inhibits caspase-1 enzymatic activity, Disulfiram (DFM) inhibits GSDMD-N cleavage. All three agents attenuate pyroptosis-driven neuroinflammation and demonstrate antiseizure efficacy in preclinical models (Created with Figdraw).

Depending on the molecular target, therapeutic strategies targeting pyroptosis fall into three mechanistically distinct categories: (i) direct inhibition of pyroptosis executioner proteins—most notably gasdermin D (GSDMD)—via covalent inhibitors, pore-blocking peptides, or genetic ablation; (ii) upstream modulation of inflammasome complexes—including NLRP3 and caspase-1—through selective small-molecule inhibitors or regulatory biologics; and (iii) broad-spectrum anti-inflammatory agents that indirectly suppress pyroptosis by reshaping the neuroinflammatory microenvironment (e.g., IL-1β-neutralizing antibodies, TNF-α inhibitors). (iv) Traditional Chinese medicine-derived compounds and natural bioactive products with pleiotropic effects (e.g., α-pinene). A comprehensive overview of current preclinical evidence, translational challenges and clinical development status for each strategy is provided below (Table 1 summarizes representative compounds; Figure 3 illustrates their molecular mechanisms of action).

**TABLE 1:** Comprehensive overview of additional drugs or molecules modulating pyroptosis.

Drug name	Target site of action	References
Donglingcao A	Inhibits NLRP3/caspase-1 pathway	Zhao et al. (2024a)
Guanidine	Inhibits TLR4/MYD88/NF-κB/NLRP3 pathway	Li et al. (2021)
α-Pinene (APN)	Inhibits ERK1/2 and NF-κB	Hashemi et al. (2025)
Baicalin (BA)	Inhibits NLRP3/GSDMD pathway	Kang et al. (2025)
Chaihu Longgu Muli Tang (CLMT)	Inhibits NLRP3 Inflammasome and caspase-1	Xia S. et al. (2021)
SIRT3	Inhibits NLRP3 Inflammasome	Sun Z. et al. (2023)
Galectin-3	Inhibits NLRP3/Pyroptosis	Sun W. et al. (2023)
PF-06650833	Inhibits NLRP3	Zhao et al. (2024b)
Glycyrrhizin	Inhibits NLRP3	Wei et al. (2025)
Triterpenoid alkaloids (TAC)	Inhibits NLRP3/caspase-1/GSDMD HMGB1/TLR4/NF-κB pathway	Qi et al. (2023)
NS8593	Inhibits NLRP3	Tong et al. (2024)
Vitexin (VT)	Inhibits P2X7R-NLRP3	Chen et al. (2025)
TRPV4	Inhibits NLRP3/caspase-1/GSDMD pathway	Liu et al. (2025)
FOXC1	Inhibits NLRP3	Yang and Yu (2025)
BmK IT2	Inhibits NLRP1/caspase-1/GSDMD pathway	Qu et al. (2026)
Biochanin A	Inhibits NLRP3	El-Sayed et al. (2023)
Roflumilast	Inhibits NLRP3/caspase 1	Fawzy et al. (2025)

### 3.1 Direct inhibition of pyroptosis executioner proteins: targeting GSDMD-mediated pore formation

GSDMD serves as the terminal executioner of pyroptosis, with its N-terminal fragment (GSDMD-NT) oligomerizing at the plasma membrane to form pores—a conserved hallmark across both canonical and non-canonical pyroptotic pathways. Pharmacological blockade of GSDMD pore formation therefore represents a highly specific therapeutic node, capable of suppressing downstream inflammatory lytic death while preserving upstream immune sensing functions—thereby minimizing off-target immunosuppressive effects (Wu et al., 2023).

Disulfiram (DFM), an FDA-approved agent for alcohol use disorder, was serendipitously identified as a covalent inhibitor of GSDMD. Hu et al. (2020) first reported that disulfiram irreversibly blocks GSDMD pore formation by covalently modifying the Cys191 residues of GSDMD, thereby abrogating pyroptosis independently of NLRP3 inflammasome or caspase-1 activity. In epilepsy research, Zhang M. et al. (2025) demonstrated that in a KA-induced epileptic murine model, disulfiram administration significantly attenuated astrocytic pyroptosis in the hippocampus—as evidenced by reduced GSDMD-N cleavage—concomitantly restored blood–brain barrier integrity via upregulation of tight junction proteins (ZO-1, claudin-5, occludin), decreased seizure frequency and severity, and ameliorated hippocampal-dependent cognitive deficits (Zhang M. et al., 2025). Furthermore, dimethyl fumarate (DMF) — an FDA-approved disease-modifying therapy for multiple sclerosis—has been shown to inhibit GSDMD oligomerization via succinylation at Cys191, thereby preventing pore assembly. Although preclinical evidence supports its mechanistic relevance to pyroptosis suppression, DMF’s antiseizure efficacy and optimal dosing regimen in epileptic models remain to be rigorously established (Ryder et al., 2022).

### 3.2 Upstream modulation of inflammasomes: targeting NLRP3 and caspase-1

Inflammasome activation—particularly that of the NLRP3 complex—represents a critical upstream regulatory node in canonical pyroptosis. Pharmacological inhibition at this level suppresses caspase-1 activation, thereby attenuating the proteolytic maturation of IL-1β and IL-18 and curtailing downstream pyroptotic cell death and neuroinflammation (Wu et al., 2025).

#### 3.2.1 NLRP3-specific inhibitors

MCC950 is a well-characterised, highly potent and selective small-molecule inhibitor of NLRP3 inflammasome with no appreciable activity against other inflammasome complexes—including NLRP1, NLRC4, and AIM2. Its target specificity enables partial preservation of innate immune surveillance by avoiding global suppression of cytokine release, thereby maintaining essential host defence functions *in vivo*. Preclinical studies consistently demonstrate robust antiseizure and neuroprotective effects of MCC950 across multiple rodent models of epilepsy.

Mechanistically, MCC950 inhibits NLRP3 inflammasome assembly, inhibits caspase-1 activation, and consequently attenuates the maturation and secretion of pro-inflammatory cytokines IL-1β and IL-18. In both pentylenetetrazole (PTZ)- and KA-induced epilepsy models, MCC950 treatment effectively alleviated seizure severity, mitigated neuronal loss, and improved cognitive function (Hong et al., 2024; Hou et al., 2024). JC124 represents another novel, structurally distinct NLRP3-specific inhibitor. A recent summary Mendelian randomisation analysis by Zhang P. et al. (2025) identified a positive genetic correlation between NLRP3 expression and epilepsy susceptibility. JC124 treatment markedly suppressed KA-induced seizures in mice, alleviated depression-like behaviours and cognitive impairment, and mitigated hippocampal neurodegeneration—including neuronal pyroptosis—as well as glial hyperactivation (microglial and astrocytic proliferation). Mechanistic investigations further revealed that JC124 directly binds to the human NLRP3 protein and exertspotent anti-neuroinflammatory and antioxidant effects in human induced pluripotent cell–derived neurons (hiPSC-neurons) (Zhang P. et al., 2025).

#### 3.2.2 Caspase-1 inhibitors

Sinomenine has been demonstrated to inhibit caspase-1 cleavage in the KA-induced model, thereby mitigating neuronal pyroptosis and exerting anticonvulsant and neuroprotective effects (Ramazi et al., 2020). Ac-YVAD-cmk—a selective, cell-permeable caspase-1 inhibitor—is currently under investigation for neurological disorders, including intracerebral haemorrhage and cognitive impairment (Zheng et al., 2023). Its neuroprotective action is mediated primarily through inhibition of the NLRP3/caspase-1/IL-1β signalling axis, consistent with shared upstream inflammasome regulation. VX-765 (also known as belnacasan) is a selective, orally bioavailable prodrug of the active caspase-1 inhibitor VRT-043198. Preclinical studies have demonstrated its anticonvulsant properties in both acute and chronic epilepsy models. Maroso et al. (2011) reported that systemic administration of VX-765 dose-dependently reduced acute kainic acid-induced seizures in mice, with significant effects observed at doses ≥50 mg/kg. In a chronic model of temporal lobe epilepsy characterized by spontaneous recurrent epileptiform activity refractory to phenytoin, repeated daily administration of VX-765 (50–200 mg/kg) significantly reduced the time spent in epileptic activity by 50%–75%, an effect that was reversible upon drug discontinuation. This anticonvulsant effect was associated with inhibition of IL-1β synthesis in activated astrocytes, whereas glial activation *per se* was not affected, supporting the specificity of the mechanism (He et al., 2020; Maroso et al., 2011). Treatment with CZL80—a novel, brain-penetrant caspase-1 inhibitor identified through structure-based drug design. In multiple acute seizure models—including the maximal electroshock (MES), pentylenetetrazol (PTZ), and amygdaloid kindled models—CZL80 demonstrates broad-spectrum antiseizure efficacy. It significantly reduces seizure severity, prolongs the latency to generalized seizures, and improves survival rates in a dose-dependent manner. Mechanistically, CZL80 directly inhibits neuronal excitability by reducing spontaneous firing frequency and increasing the rheobase current required to elicit action potentials. Notably, CZL80 enhances inhibitory neurotransmission by increasing the amplitude of spontaneous inhibitory postsynaptic currents (sIPSCs) without affecting excitatory transmission, a mechanism distinct from conventional antiseizure medications that primarily target ion channels or GABA receptors. The antiseizure effects of CZL80 are abolished in caspase-1 knockout mice, confirming its target specificity. Chronic administration of CZL80 for 3 weeks does not impair locomotor activity, indicating a favorable safety profile. Collectively, these findings position CZL80 as a promising caspase-1-targeted antiseizure agent with translational potential (Tang et al., 2020; Wang et al., 2024).

### 3.3 Broad-spectrum anti-inflammatory agents and multi-target therapeutic strategies

Beyond pharmacological agents that directly inhibit pyroptosis, several broad-spectrum anti-inflammatory agents exert indirectly modulatory effects on this pathway by reshaping the neuroinflammatory milieu. Notably, some of these compounds have demonstrated clinically relevant antiseizure activity in human studies. Edaravone—a potent free radical scavenger and approved therapeutic for amyotrophic lateral sclerosis (ALS) and acute ischemic stroke—has shown promise in epilepsy models. In a recent study using a rat model of pilocarpine-induced status epilepticus, Noriega-Ruiz et al. (2025) observed that edaravone administration significantly downregulated NLRP3 inflammasome expression and ameliorated reactive gliosis, as evidenced by reduced morphological activation of both microglia and astrocytes. These findings support the hypothesis that redox-modulating agents may suppress pyroptotic pathway by inhibiting the NLRP3 inflammasome, thereby providing a potential avenue for the indirect upstream regulation of neuronal pyroptosis (Noriega-Ruiz et al., 2025).

### 3.4 Traditional Chinese medicine-derived compounds and natural bioactive products

Zhang et al. (2024) systematically reviewed various naturally derived compounds with demonstrated antiseizure efficacy mediated through modulation of the pyroptosis pathway, including methyl salicylate (commonly referred to as “wintergreen oil” or “wintergreen A,” targeting the NLRP3/caspase-1 axis), astragaloside IV (not astragaloside A; correcting nomenclature and specifying the predominant bioactive isomer in Astragalus membranaceus, which modulates the TLR4/NF-κB/NLRP3 cascade), α-pinene (inhibiting the ERK1/2 and NF-κB pathways), baicalin (suppressing NLRP3-dependent GSDMD cleavage and pore formation), and multi-herb formulations such as Chaihu Longgu Muli Tang (CLMT) (Zhang et al., 2024). Han et al. (2024) developed resveratrol-loaded cationic liposomal nanoparticles (C-LIPS/RV), which significantly attenuated seizure burden and rescued cognitive deficits in a murine model of post-traumatic epilepsy—mechanistically via ROS scavenging, suppression of IL-1β and IL-18 maturation, and inhibition of neuronal pyroptosis. Although these multi-component, multi-target interventions hold promise for synergistic neuroprotection and disease modification, rigorous phytochemical standardisation, deconvolution of active constituents, mechanistic validation in human-relevant models, and comprehensive preclinical safety profiling remain critical knowledge gaps warranting further systematic investigation.

### 3.5 Clinical development status and safety assessment of key pyroptosis-targeting therapeutics

Although pyroptosis-targeting agents have demonstrated promising antiseizure efficacy in preclinical epilepsy models, their translation from bench to bedside remains hindered by several critical challenges. The following section provides a critical analysis across three key dimensions: blood–brain barrier permeability, clinical trial status, and safety profile.

#### 3.5.1 Blood–brain barrier penetration: a key obstacle to CNS targeting

The blood–brain barrier constitutes the main physical barrier impeding drug delivery to the CNS. Most small-molecule pyroptosis inhibitors—including MCC950, VX-765, and disulfiram—exhibit limited blood–brain barrier permeability, representing a major bottleneck in their clinical translation. The C-LIPS/RV, developed by Han et al. (2024), attenuated pyroptosis and ameliorated cognitive deficits in a murine model of post-traumatic epilepsy by scavenging ROS and suppressing IL-1β/IL-18 maturation. Collectively, these findings suggest that nanodelivery systems may serve as an effective strategy for overcoming the blood–brain barrier permeability limitation inherent to pyroptosis-targeting agents.

#### 3.5.2 MCC950: potent NLRP3 inhibition with clinical development halted by hepatotoxicity

MCC950 is the prototypical, highly selective small-molecule inhibitor of the NLRP3 inflammasome and has consistently demonstrated robust antiseizure efficacy across various epilepsy models. However, in phase II clinical trials, the drug induced elevated serum liver enzyme levels, leading to the termination of the clinical study (Mangan et al., 2018). Echanistic studies indicate that this toxicity is likely attributable to structural features—including the furan ring and the sulfonylurea moiety—which collectively compromise both safety and oral pharmacokinetic performance.

Notably, the sulfonylurea group exhibits marked instability under gastric acidic conditions, resulting in low and variable oral bioavailability and suboptimal drug-like properties (Zheng et al., 2024; Feng et al., 2024).

#### 3.5.3 VX-765: a phase II–evaluated caspase-1 prodrug with favorable safety but inconclusive antiseizure efficacy

VX-765 is an orally bioavailable prodrug of the caspase-1 inhibitor VRT-043198, designed to overcome the poor pharmacokinetic properties of its active metabolite (Maroso et al., 2011). In a Phase II, randomised, double-blind, placebo-controlled proof-of-concept trial enrolling 60 adults with refractory partial focal-onset epilepsy, VX-765 demonstrated a favorable safety and tolerability profile: treatment-emergent adverse events—including headache, dizziness, fatigue, and gastrointestinal disturbances—were predominantly mild to moderate in severity and resolved without intervention. However, the trial failed to achieve its primary efficacy endpoint, as there was no statistically significant difference between the VX-765 and placebo groups in either seizure frequency reduction or responder rate. These results indicate that caspase-1 inhibition may require longer-duration exposure or optimized dosing regimens to elicit clinically meaningful antiseizure effects—highlighting the need for mechanistically informed trial design in future development programs (Bialer et al., 2013).

#### 3.5.4 Disulfiram: a repurposing candidate for pyroptosis inhibition with significant safety considerations

Disulfiram is a long-standing FDA-approved agent (1948) for the management of chronic alcohol use disorder. Emerging evidence demonstrates that disulfiram directly inhibits GSDMD pore formation, offering a mechanistically sound rationale for repurposing this drug for epilepsy and other neuroinflammatory conditions. However, its clinical redeployment necessitates rigorous safety evaluation, given its well-documented risk profile, including hepatotoxicity, neuropathy, and psychiatric disturbances. A retrospective analysis of real-world adverse event reports from the FDA Adverse Event Reporting System (FAERS), covering the fourth quarter of 2002 through the third quarter of 2023, identified 508 cases in which disulfiram was the primary suspect drug.

These reports encompassed 104 preferred terms distributed across 25 System Organ Classes (SOCs). The most frequently reported adverse events were: hepatobiliary disorders, neurological disorders psychiatric symptoms, and cardiac toxicity (Luo et al., 2024).

## 4 Conclusions and future directions

Pyroptosis has emerged as a critical pathophysiological mechanism in epilepsy, functioning as a molecular bridge that couples neuroinflammation with programmed cell death and neuronal hyperexcitability. As synthesized in this review, accumulating evidence reveals cell-type-specific execution of pyroptosis: astrocytic GSDMD activation compromises blood–brain barrier integrity; neuronal pyroptosis engages the TRPM7/ROS/JAK2/STAT3 signaling axis and intersects with NLRP3 inflammasome activation and mitophagy; and microglial pyroptosis amplifies neuroinflammation. Together, these interconnected processes established a self-sustaining feedback loop that lowers seizure thresholds and promotes epileptogenesis. The recent conceptualization of PANoptosis—the coordinated, inflammatory cell death pathway integrating pyroptosis, apoptosis, and necroptosis in epileptic neurons—further highlights the multifaceted regulation of cell death in epilepsy. This paradigm shift has direct therapeutic implications: concurrent activation of multiple death pathways suggests that monotherapeutic targeting of a single effector may be insufficient to halt disease progression.

The translational landscape for pyroptosis-targeting therapies has advanced considerably, with several critical insights emerging from recent clinical and preclinical studies. The caspase-1 inhibitor VX-765 demonstrated an acceptable safety profile in Phase II trials for refractory epilepsy; however, its delayed therapeutic onset suggests that extended treatment durations maybe necessary to fully evaluate efficacy. Preclinically, the GSDMD inhibitor disulfiram shows promise in preserving blood–brain barrier integrity and reducing seizure severity. Its repurposing potential, however, is constrained by well-documented hepatotoxicity and alcohol interaction risks, underscoring the need for stringent patient selection and monitoring. The clinical discontinuation of MCC950 due to dose-dependent hepatotoxicity provides a crucial reminder that potent preclinical efficacy does not guarantee clinical success, emphasizing the importance of integrated safety assessment early in drug development. Novel delivery strategies—such as resveratrol-loaded nanoliposomes—offer a promising approach to enhance brain bioavailability while mitigating systemic exposure and toxicity.

Despite substantial progress, several critical knowledge gaps persist. First, the functional contributions of non-canonical pyroptosis pathways—including caspase-4/5/11, GSDME, and GSDMB—to epileptogenesis remain unvalidated; current evidence is limited to correlative expression changes in gene or protein expression, and direct causal inference requires cell-type-specific genetic perturbation models. Second, the recently identified LCN2/NLRP3 axis—linking astrocyte pyroptosis to impaired mitochondrial transfer between glia and neurons—represents a promising therapeutic target, yet its mechanistic underpinnings require rigorous validation across diverse epilepsy models, including genetic, acquired, and chronic seizure models. Third, compensatory crosstalk among pyroptosis, apoptosis, and necroptosis—exemplified by findings that GSDMD knockdown can paradoxically induce caspase-3-dependent apoptosis—necessitates systematic investigation to anticipate and mitigate unintended consequences of pathway-selective inhibition. Fourth, most preclinical studies rely on acute chemically induced models (e.g., kainic acid or pilocarpine), whereas validation in chronic epilepsy models and genetically defined epilepsy models is urgently warranted to improve translational relevance. Fifth, robust predictive biomarkers for patient stratification are still lacking; peripheral candidates—including plasma IL-1β, IL-18, caspase-1 activity, and serum Pannexin-1—merit prospective clinical evaluation in well-phenotyped cohorts.

Addressing these challenges necessitate multifaceted, mechanism-informed strategy. First, structure-guided drug design should prioritize the development of brain-penetrant, isoform-selective inhibitors that precisely target pyroptotic effectors—such as GSDMD or caspase-1—without perturbing functionally related proteases (e.g., caspase-3, caspase-8). Second, advanced delivery platforms—including nanoparticle-based carriers and receptor-mediated transcytosis systems—offer potential to enhance CNS bioavailability while minimizing off-target systemic exposure. Third, clinical trial designs must accommodate the delayed therapeutic onset characteristic of pyroptosis inhibitors by incorporating extended treatment durations and delayed efficacy endpoints to ensure robust assessment of disease-modifying effects. Finally, rationally designed combination therapies—simultaneously engaging complementary cell death pathways—may be essential to circumvent compensatory crosstalk and achieve durable antiseizure efficacy.

In conclusion, targeting pyroptosis represents a promising disease-modifying strategy for the approximately one-third of individuals with epilepsy who are refractory to current antiseizure medications. Realizing this potential hinges on rigorious preclinical validation in models that faithfully recapitulate the chronicity, comorbidities, and circuit-level pathophysiology of human epilepsy. It further demands the development of advanced drug delivery platforms—capable of circumventing blood–brain barrier restrictions while ensure target engagement in relevant neural and glia cell populations—and clinical trial designs that explicitly account for the spatiotemporally dynamic and cell-type-specific nature of pyroptotic signaling in the epileptic brain. The field has evolved from initial descriptive observations to mechanistic dissection and early-phase clinical evaluation; sustained, interdisciplinary efforts will be essential to translate this knowledge into effective, precision therapies for patients with drug-resistant epilepsy.
